# Mechanisms and applications of N-Methyl-N’-nitro-N-nitrosoguanidine in animal tumor models: current situation and challenges

**DOI:** 10.3389/fonc.2025.1681270

**Published:** 2025-10-23

**Authors:** Xiyan Zhang, Yupei Xu, Junwen Cao, Tong Li, Jiaqi Wang, Jingna Tao, Liju Zhang, Zhihong Li

**Affiliations:** ^1^ Department of Gastroenterology, Dongzhimen Hospital, Beijing University of Chinese Medicine, Beijing, China; ^2^ Department of Nephrology, First Teaching Hospital of Tianjin University of Traditional Chinese Medicine, Tianjin, China; ^3^ National Clinical Research Center for Chinese Medicine Acupuncture and Moxibustion, Tianjin, China; ^4^ Department of General Medicine, Eighth Medical Center, Chinese People's Liberation Army (PLA) General Hospital, Beijing, China

**Keywords:** MNNG, cancer, animal models, gastric cancer, multi-organ carcinogenicity

## Abstract

The worldwide health and economic burden of cancer is substantial, necessitating urgent, focused prevention and treatment strategies. The investigation of cancer animal modeling techniques is particularly critical. N-methyl-N’-nitro-N-nitrosoguanidine (MNNG), a nitrosamine carcinogen, is extensively utilized in the development of several tumor animal models due to its ability to replicate the natural onset of cancer. Nonetheless, MNNG exhibits a propensity for multi-organ carcinogenesis; yet, this aspect remains undiscussed. The MNNG model exhibits distinct characteristics depending on the route of administration, yet it also presents inherent limitations such as toxicity, environmental contamination, and inconsistent modeling outcomes. These issues necessitate standardized protocols to refine the model, ensuring it meets the criteria for efficient and precise tumor induction while adhering to animal welfare principles. This study examines the current applications of MNNG in gastric cancer models and models of other organs, its carcinogenic mechanisms, translational relevance to human tumors, and practical application features, with a particular focus on its use in gastric contexts. Furthermore, it summarizes and compares the advantages and disadvantages of various MNNG administration routes, as well as contrasts its carcinogenic properties with those of other chemical inducers.Through the examination of drug administration routes, dosage effects, combined modeling strategies, and model specificity, we endeavored to identify effective methods to enhance the specificity of target organs by optimizing the administration approach (local exposure, integration of advanced detection technologies with auxiliary factors). Furthermore, we encourage researchers to disclose negative results, as this practice helps improve model stability and accuracy, reduces research costs, and aligns with animal welfare guidelines.Experimental animals are crucial in scientific study. Future investigations must develop standardized protocols to minimize non-target organ damage and examine the interaction mechanisms between these animals and the tumor microenvironment.

## 1 Introduction

Cancer continues to pose a significant global public health challenge (1). Examining cancer pathophysiology and formulating prevention measures are fundamental objectives in oncology research, indicating the essential requirement for suitable tumor models. N-Methyl-N’-nitro-N-nitrosoguanidine (MNNG), a nitrosoamine carcinogen, is extensively utilized as a chemical mutagen in animal cancer models (2). It accurately replicates the high-risk factor associated with excessive nitrite consumption in daily life and induces tumors through direct contact. Adenocarcinoma is the predominant tumor type associated with mutagenesis, commonly utilized in animal models of stomach adenocarcinoma, hence offering an optimal experimental framework for investigating the cancer mechanisms and therapeutic target development induced by nitrite (3).

In accordance with the 3R principles of animal ethics (4), Replacement should be the first and foremost consideration when planning related studies. As a widely used chemical carcinogen for establishing tumor models, MNNG often involves complex *in vivo* microenvironments and systemic disease progression, which are difficult to fully replicate using *in vitro* cell cultures. Although recent years have seen attempts to induce tumors using MNNG in 3D organoid models (5), the high cost and technical immaturity of these systems mean that animal studies remain one of the primary approaches for investigating nitrite-induced primary tumors. Therefore, under current technological constraints, upholding animal ethics relies critically on the implementation of Reduction and Refinement.By adopting more scientific experimental designs, researchers can maximize the value derived from each animal, reduce the total number of animals used, and minimize the pain and stress experienced by animals throughout the study.

Nonetheless, a significant limitation of MNNG in tumor model establishment is its relatively low specificity. Previous studies have shown that MNNG promotes carcinogenesis not only in target organs but also in non-target sites (6), which increases experimental cost and uncertainty. However, its broad systemic effects across various organ systems remain poorly characterized. Moreover, MNNG administration protocols vary considerably across different tumor models, and even within the same animal species, standardized methodologies are lacking. MNNG is an extremely potent carcinogen, and its use entails significant exposure risks as well as potential harm to the environment.It is therefore essential to comprehensively evaluate the strengths and limitations of various modeling approaches, promote adherence to the Reduction and Refinement principles, and ensure that ethical considerations for animal welfare are fully integrated without compromising scientific objectives.

## 2 Research landscape of MNNG and its implications for cancer development

MNNG, a nitrosourea compound, mimics dietary nitrite intake in humans and is widely used to model gastric mucosal carcinogenesis (7). N-nitrosamines are strongly associated with various cancers. Recent studies have extensively utilized MNNG-induced animal models to investigate tumorigenesis.MNNG enables the establishment of both *in vitro* and *in vivo* models for esophageal, uterine, lung, and colon cancers (8). Furthermore, MNNG drives tumor development by dysregulating multiple signaling pathways, including cellular immunity, oxidative stress, inflammatory response, glycolysis, apoptosis, autophagy, and proliferation (9–11). Through its multi-mechanism, multi-stage complex network, MNNG recapitulates key molecular events in human carcinogenesis and provides a valuable experimental model for clinical translation.Based on the MNNG model, numerous phytochemicals with potential for cancer prevention have been screened, and their therapeutic targets have been explored, serving clinical cancer treatment (12).

## 3 Mechanisms of tumorigenesis induced by MNNG

**Figure 1:**
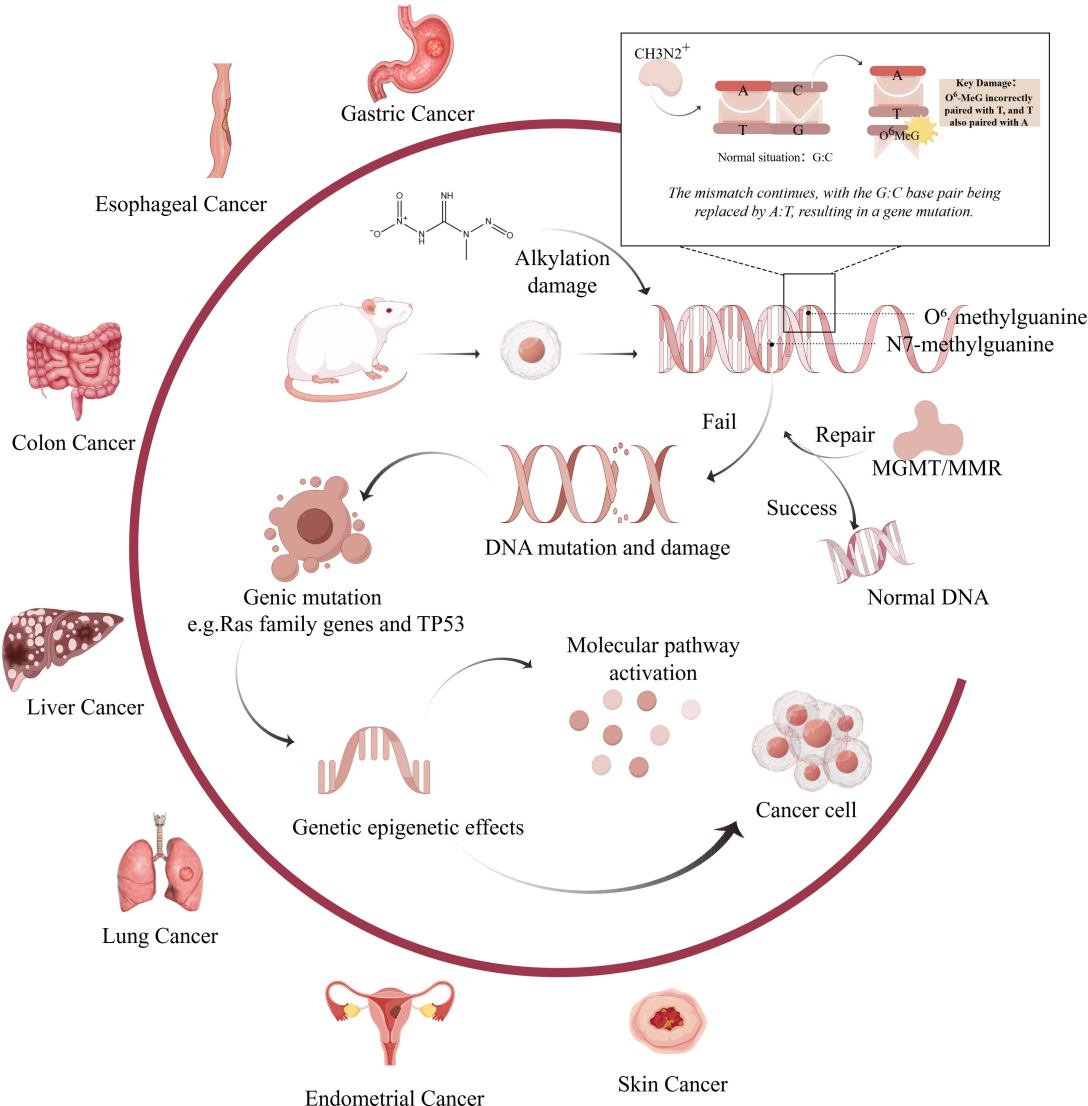
Mechanism of carcinogenic effects of MNNG on various organs.(Created by figdraw,ID : WPIWUff28f). MNNG directly alkylates DNA bases, primarily forming mutagenic adducts such as O^6^-methylguanine (O^6^-MeG) and N7-methylguanine (N7-MeG). Misincorporation of thymine opposite O^6^-MeG during replication results in G→A transition mutations. Inadequate repair of these lesions by mechanisms such as O^6^-methylguanine-DNA methyltransferase (MGMT) or mismatch repair (MMR) systems leads to persistent DNA damage. Chronic damage contributes to the activation of oncogenes (e.g., Ras family genes) and inactivation of tumor suppressors (e.g., TP53), ultimately promoting tumor development in various organs including the stomach, esophagus, colon, liver, lung, and endometrium.

**Figure 2:**
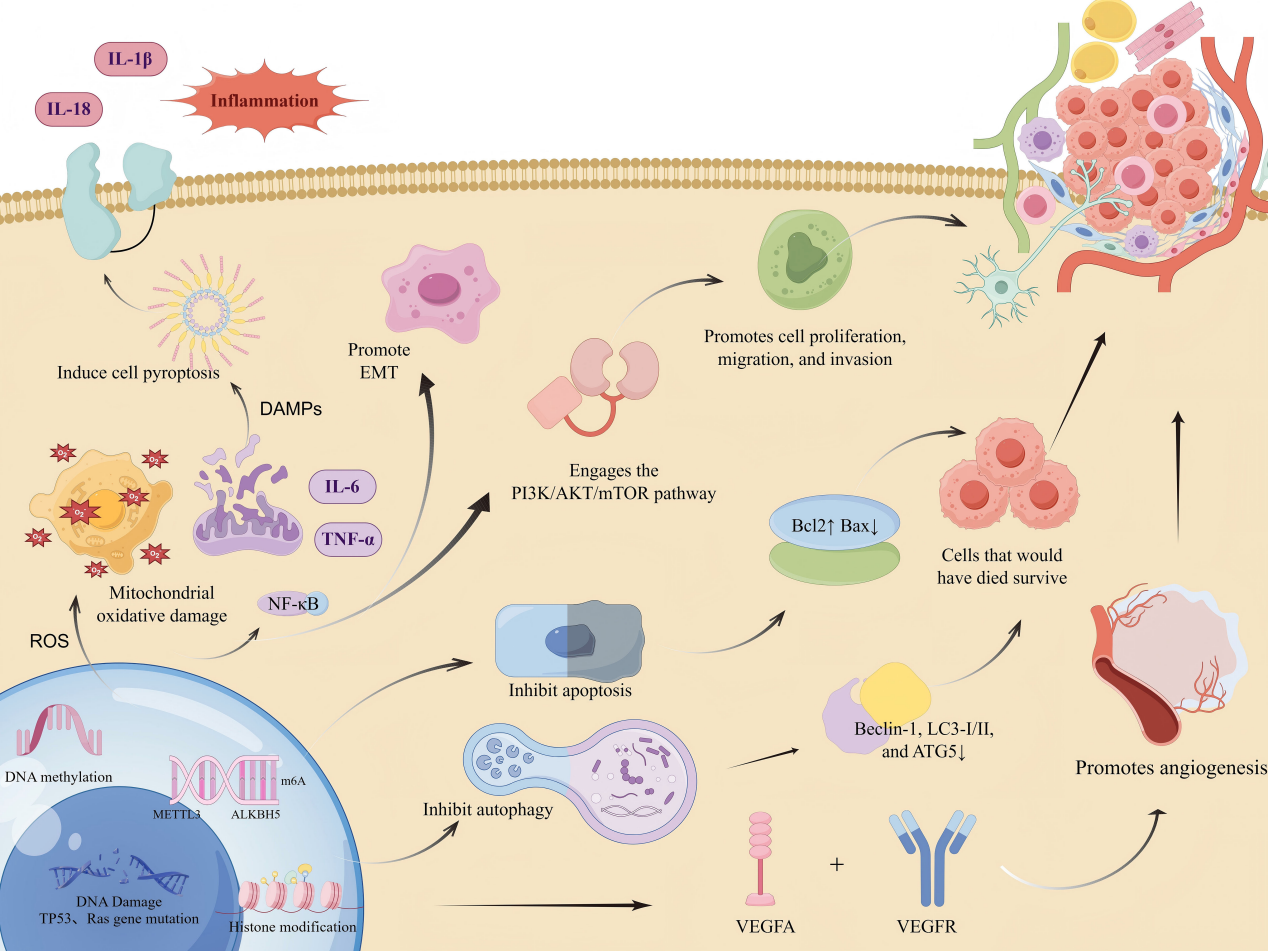
Molecular mechanisms underlying MNNG-promoted tumorigenesis.(Created by figdraw,ID : IPAYSa222a). MNNG induces DNA damage and mutations in key genes such as TP53 and Ras. It also modulates gene expression through epigenetic mechanisms including DNA methylation, histone modifications, and m6A RNA methylation. MNNG induces a sharp increase in reactive oxygen species (ROS), leading to oxidative stress and mitochondrial damage. This, in turn, triggers the release of damage-associated molecular patterns (DAMPs). The DAMPs then activate the NLRP3 inflammasome, which promotes caspase-1-mediated pyroptosis and the cleavage and maturation of pro-inflammatory cytokines, resulting in the secretion of mature IL-1β and IL-18.Concurrently, elevated ROS activates the NF-κB signaling pathway. NF-κB activation drives the transcription of pro-inflammatory genes (including those encoding pro-IL-1β and pro-IL-18) and epithelial-mesenchymal transition (EMT)-related genes, thereby further amplifying the inflammatory response and promoting EMT.These effects collectively cause dysregulation of critical signaling pathways, including PI3K/AKT/mTOR, resulting in suppressed apoptosis and autophagy. Key markers such as Bcl-2, Bax, Beclin-1, LC3, and ATG5 are altered. Moreover, MNNG enhances cell proliferation, migration, invasion, and angiogenesis via VEGFA.

The incidence of MNNG-induced tumors is closely associated with its carcinogenic mechanism. The carcinogenic properties of MNNG were initially documented by Sugimura and Fujimura, who effectively induced glandular stomach tumors in rats via prolonged exposure to MNNG in drinking water (13, 14). Since then, research into the tumorigenic processes of MNNG has advanced dramatically. It can induce carcinogenic effects directly, without the need for bioenzyme metabolism, indicating that exposure to MNNG elevates cancer risk and is more likely to affect non-target organs. **Figures 1**, **2** illustrates the schematic diagram of its mechanism.

### 3.1 DNA alkylation injury

MNNG is a powerful, direct-acting mutagenic nitroso chemical. Its reactive metabolites (e.g., methyldiazonium ions, CH_3_N_2_^+^) directly interact with DNA bases, resulting in alkylation damage and subsequent gene alterations that promote carcinogenesis.The primary targets of DNA alkylation are the O6 and N7 positions of guanine, as well as the N3 position of adenine.Following MNNG exposure, the primary adduct identified in double-stranded DNA was N7-methylguanine (N7-MeG) at 67%, along with the minor adducts N3-methyladenine (N3-MeA) (12%) and O^6^-methylguanine (O^6^-MeG) (7%) (15). The O^6^-methylguanine (O^6^-MeG) adduct formed at the O6 position is the most mutagenic lesion and represents a key form of DNA damage induced by MNNG (16). During DNA replication, DNA polymerase misincorporates thymine (T) opposite O^6^-MeG, rather than cytosine (C), which is the correct partner for guanine.Normal cells typically contain a greater number of G-C base pairs than malignant cells, a phenomenon attributed to the mispairing of O^6^-MeG with thymine, which leads to a reduction in methylatable cytosine residues. Subsequently,O^6^-MedG can induce sister chromatid exchanges(SCE), chromosomal aberrations, and further lead to double-strand breaks, thereby promoting genetic mutations and tumorigenesis (17, 18). Furthermore, the key MNNG-induced DNA adduct O^6^-MeG contributes to mutagenesis by activating proto-oncogenes or inactivating tumor suppressor genes.Mutations in Ras family genes, such as H-Ras at codons 12 and 13, result in persistent proliferative signaling (9, 10). Mutations in tumor suppressor genes, including TP53 ans P53, can lead to the inactivation of tumor suppression,hence promoting carcinogenesis (19, 20).

### 3.2 Failure of the DNA repair mechanism

Approximately 97% of N7-MeG adducts are eliminated from pyloric mucosa within 48 hours following MNNG exposure, possibly attributable to active base excision repair (BER) (21). Base excision repair (BER) is primarily responsible for repairing base alkylation damage. DNA glycosylases recognize and excise the damaged bases, thereby initiating subsequent cleavage and ligation steps.The repair of O^6^-MedG is contingent upon the O6-methylguanine-DNA methyltransferase(MGMT) (16, 22). In gastric cancer, early upregulation of MGMT promotes DNA damage repair; however, hypermethylation of its promoter at a later stage leads to reduced MGMT expression (18, 23, 24). Inhibition of MGMT function induces G:C to A:T mutations in the tumor suppressors p53 and PTEN, contributing to human carcinogenesis (25). The mispaired O^6^-MeG:T lesion is recognized by the mismatch repair (MMR) system. This is supported by the observed upregulation of MMR-related proteins MSH2 and MSH6 in MNNG-treated cells (26). Deficiencies in this essential DNA repair mechanism can lead to the accumulation of mutations, thereby promoting tumor development (27, 28).However, this repair process also could be fatal, as it can lead to DNA double-strand breaks (29).

### 3.3 Affecting genetic characteristics of genes

MNNG induces not only genotoxic effects (gene mutations) but also profoundly influences gene expression profiles through epigenetic mechanisms, thereby driving tumorigenesis. DNA methylation represents a well-characterized epigenetic feature of MNNG exposure, characterized by the concomitant occurrence of global hypomethylation and localized promoter hypermethylation of specific genes, leading to oncogene activation and tumor suppressor gene silencing (30). For instance, hypermethylation of the tumor suppressor gene p16 promoter and hypomethylation of the oncogene hTERT have been documented in MNNG-induced carcinogenesis (31, 32). Additionally, MNNG can modulate the epigenome by upregulating phosphorylation of histone H3 at serine 10 and 28 (H3S10p, H3S28p) and downregulating acetylation of histone H4 at lysine 16 (H4K16ac) (33). Furthermore, METTL3 can promote gastric carcinogenesis by activating the METTL3/m6A/miR-1184 axis via an m6A-dependent mechanism, thereby interfering with the miR-1184/TRPM2 signaling pathway (34).Recent findings indicate that the demethylase ALKBH5, which regulates ZKSCAN3 expression via N^6^-methyladenosine (m^6^A) modification, activates VEGFA transcription and facilitates MNNG-induced gastric cancer cell migration, invasion, cancer stem cell (CSC) generation, vasculogenic mimicry (VM), and ultimately, gastric cancer progression (13).

### 3.4 Influence the molecular mechanism of tumor

Exposure to MNNG can induce an oxidative stress response. Following MNNG treatment, levels of reactive oxygen species (ROS) increase, while the activity of antioxidant enzymes SOD, CAT, and GSH-Px significantly decreases in both blood and gastric tissues (35). This enhances mitochondrial oxidative damage and promotes cell division, which is one of the primary mechanisms of mutagenesis.Concurrently, this process activates DAMPs, triggering the NLRP3 inflammasome and amplifying the inflammatory response. It is widely recognized that MNNG exposure induces inflammation in the gastric mucosa. In tissues subjected to chronic exposure, the expression levels of inflammatory factors such as IL-6, IL-18, IL-1β, TNF-α, and NF-κB are elevated (36). Similarly, the levels of Gasdermin D (GSDMD), NLR family pyrin domain containing 3 (NLRP3), and Caspase-1 are also upregulated (37), indicating that pyroptosis is indeed involved in the inflammatory burst. This aligns with the progression of inflammation-to-cancer transformation.

O^6^-MeG secondarily induces DNA double-strand breaks and triggers apoptosis through upregulation of p53 and Fas/CD95/Apo−1, accompanied by decreased Bcl-2 and activation of caspase-9 and caspase-3 (38, 39). However, in the gastric cancer microenvironment, increased expression of Bcl-2 along with reduced levels of Bax, Bim, caspase-8, and caspase-3 indicates suppressed apoptosis (40). The survival of cells that were destined to die,which may accelerate tumor progression.

MNNG also leads to accumulation of p62 and engages the PI3K/AKT/mTOR pathway downstream (41), contributing to malignant processes such as cell proliferation, migration, and invasion. MNNG can suppress normal autophagy and promote the epithelial-mesenchymal transition (EMT) and cell proliferation. Long-term exposure to MNNG reduces the expression of autophagy-related proteins including Beclin-1, LC3-I/II, and ATG5 (42).

Furthermore, following MNNG intervention, regulation of MMP2, MMP9, VEGF, VEGFR1, TIMP-2, and RECK promotes angiogenesis (43), tumor invasion, and metastasis. Many phytochemicals have been found to exhibit preventive effects against tumor formation in MNNG-induced tumor models. Tumor development is multidimensional and involves synergistic activity across multiple pathways; the mechanisms underlying MNNG-induced carcinogenesis are not yet fully elucidated and remain under active investigation.

## 4 Utilization of MNNG in various tumor models

### 4.1 MNNG in gastrointestinal models

#### 4.1.1 Animal models associated with gastric cancer

In gastric cancer models, MNNG predominantly induces well-differentiated intestinal-type adenocarcinomas (44, 45). Beyond the previously outlined mechanisms of MNNG carcinogenesis, there are specific characteristics in the mechanism by which MNNG results in gastric adenocarcinoma. It selectively alkylates pyloric gland cells (46), leading to lesions primarily confined to the pyloric region (47, 48), potentially attributable to enhanced carcinogen accessibility to proliferative cells in the gastric antrum (49). Research indicates that the prevalence of O^6^-MedG-positive cells diminishes systematically from the pylorus to the corpus, forestomach, duodenum, and esophagus (50).

Male rats demonstrate elevated tumor induction rates (up to 88%) compared to female rats (51–53) and are favored. Strains exhibiting increased vulnerability including Wistar-Kyoto, Wistar, Sprague-Dawley (SD), and ACI rats (54–56). Tumor induction rates are often elevated in rats younger than 12 weeks and weighing < 120 g.

##### 4.1.1.1 MNNG-induced models: single-agent approach

MNNG can be delivered through free drinking and intragastric gavage in the development of gastric cancer models. Free drinking refers to rats ingesting MNNG at a certain concentration dissolved in drinking water ad libitum. The concentration of MNNG free drinking water, the administration period and the success rate of modeling are shown in **Table 1**. Generally, tumors generated by MNNG require an extended period for development. MNNG must be administered in drinking water for a duration of 8-10 weeks to effectively begin adenoma developmen (66). Within 30 weeks of administration, there may be a positive correlation between MNNG induction success and time of administration.Notably, prolonging exposure to 40 weeks at this concentration unexpectedly decreased the incidence to 43.8% (65).

**Table 1:** Overview of MNNG free drinking water technology in gastric cancer model.

MNNG dosing groups	Dosage of MNNG	Duration (Wks)	Incidence (%)	Co-factor	Animal strains	Repeated verification	Quote
Low- dosage	25 μg/mL	25	60	None	Wistar male rats	Have	(57)
	25 μg/mL	25	30	None	Wistar male rats	Have	(58)
	25 μg/mL 50 μg/mL	32	22.2 29.6	None	Wistar male rats	Have	(59)
	50 μg/mL	25	72	None	Wistar male rats	Have	(60)
	50 μg/mL	25	30	None	Wistar male rats	Have	(61)
Middle-dosage	83 μg/mL	26	50	None	Wistar male rats	Not	(62)
	83 μg/mL	24	30	None	Wistar male rats	Have	(63)
	83μg/mL	8; 16; 26	37.5 43 28	None	Wistar male rats	Not	(64)
High-dosage	100μg/mL	8	40.6	None	Sprague-Dawley male rats	Have	(65)
	100μg/mL	8	26.7	None	Wistar male rats	Not	(66)
	100μg/mL	8	10	None	Wistar male rats	Not	(67)
	100μg/mL	17	60	Diet with 8%NaCl	Wistar male rats	Not	(45)
	100μg/mL	24	73.3	None	Wistar male rats	Not	(68)
	100μg/mL	25	80	None	Wistar male rats	Not	(69)
	100μg/mL	28	84.2	None	Wistar male rats	Not	(70)
	100μg/mL	30	64.3	None	Sprague-Dawley male rats	Not	(71)
	100μg/mL	32	44.4	None	Wistar male rats	Not	(59)
	100μg/mL	40	43.8	0.9%Nacl	Wistar male rats	Not	(65)

It has been reported that the incidence of MNNG-induced tumors at 32 weeks was actually lower than that at 24 weeks (32). Therefore, we consider 24–30 weeks to be a relatively ideal treatment window when administering MNNG via drinking water.Although high doses of MNNG can induce a higher tumor incidence, increasing its concentration does not lead to a proportional rise in modeling success. This is likely because rats have a sensitive sense of smell, and higher concentrations of MNNG may reduce their water intake.A concentration of 100 μg/mL may be an optimal choice for drinking water administration, though further experimental validation is needed to confirm this hypothesis.

However, we also observed substantial heterogeneity in tumor induction rates even under repeated experiments using the same administration protocol. Although higher doses of MNNG generally lead to relatively higher success rates in tumor induction, significant variability remains across studies.

Certain scholars have noted that the ingestion of MNNG at a concentration of 100 mg/L through drinking water results in an actual cumulative intake of 150 mg to 250 mg, which is insufficient to promote tumor formation; the cumulative MNNG dose required for tumor induction is 300 mg (72). These conflicting observations may indicate considerable instability in the modeling process when MNNG is administered via drinking water, likely related to its susceptibility to degradation under room temperature conditions, which could represent a potential limitation. Furthermore, the lack of reported details in some experimental datasets,such as the age and weight of the animals, housing conditions, diet and water source, manufacturer and storage conditions of MNNG, as well as mortality rates during the experiment,has also hindered a standardized analysis.

Unrestricted drinking modeling more accurately reflects the normal pathological progression and can generate cancer models that align with the adenocarcinoma development pattern observed in humans. It possesses significant reference value and serves as an exemplary modeling method. This method is currently prevalent, and its value resides in its straightforward execution, which can successfully mitigate harm inflicted by mechanical procedures such as surgery or intragastric gavage on rats, hence decreasing the mortality rate. Nevertheless, the free drinking method possesses certain drawbacks. MNNG is light-sensitive and necessitates storage at 2-8°C; while employing shade treatment, elevated interior temperatures may potentially lead to gradual degradation at ambient temperature. Unrestricted access to drinking water does not ensure the daily water consumption of each rat, and elevated concentrations of MNNG inhibit water intake (6, 73). Residual MNNG effluent presents environmental disposal difficulties. Extended modeling durations elevate the possible dangers of operator exposure.

Intragastric gavage is a frequently employed modeling technique. The drug concentration is often established at 150-250 μg/mL and the compound is delivered directly into the stomach. The induction of tumors is positively correlated with the dosage. Elevating gavage concentration from 50 mg/kg to 100 mg/kg augmented the incidence of forestomach squamous cell carcinoma from 21% to 30%, and glandular stomach dysplasia to 52%. The success rate of model construction by the intragastric gavage approach is elevated, and it is occasionally combined with NaCl to enhance the success rate. Two doses of 200 mg/kg/bw resulted in a 100% success rate (74–77). This page compiles data on commonly utilized gavage doses and their high success rates in recent years for researchers’ reference, as illustrated in **Table 2**.

**Table 2:** Overview of MNNG Intragastric Gavage Technique for Gastric Cancer Models.

MNNG Dose & Schedule	Co-Factors	Incidence (%)	Repeated verification	Animal strains
250 mg/kg/bw, single dose	None (78)	82.4	Not	BD-VI male rats
200 mg/kg/bw, Days 0 & 14	1ml NaCl daily × 6w (77); 1ml sat. NaCl 3x/wk × 4w (74); 1mg sat. NaCl 2x/wk × 3w (79); None (80)	100	Have	Wistar male rats
150 mg/kg/bw, Days 0 & 14	S-NaCl every 3 days × 3w (81)	100	Have	Wistar male rats
150 mg/kg/bw, 3 doses (2wk apart)	None (82, 83)	100	Have	Wistar male rats

The acute LD_50_ for MNNG in 10% DMSO administered via gavage is 90 mg/kg (75), and a dosage of 100 mg/kg resulted in 52% immediate death. It should be noted that gavage administration involves high-dose delivery over a short period, which may lead to acute toxicity and mortality in animals. Although this risk appears to be closely linked to the solvent used, employing aqueous or olive oil solutions can significantly reduce such potential harm.

Intragastric gavage models demonstrate an elevated incidence of forestomach tumors (12%) (84, 85), potentially rendering them suboptimal for studies on glandular gastric adenocarcinoma. This may be attributed to the proximity of the specifications and maximum length of the gastric needle to those of the forestomach, complicating access to the anatomical location of the glandular stomach. The intragastric gavage approach offers the benefit of precise regulation of daily MNNG intake in experimental animals, hence enhancing the model’s success rate. MNNG solutions are freshly created, reducing waste and decreasing environmental and operator exposure. Nonetheless, the drawback of intragastric gavage is that it necessitates a high level of expertise from the experimental team. Prolonged, high-dose gavage administration is likely to have deleterious effects, including esophageal damage and gastrointestinal distension in rats, potentially leading to increased mortality (86).

##### 4.1.1.2 MNNG-induced models: combination approach

Owing to the protracted lengthand inconsistent efficacy of MNNG in isolation, composite modeling techniques have gained prominence in recent years, especially in the investigation of the therapeutic mechanisms of natural medicines targeting stomach precancerous conditions (87). The efficacy of modeling is generally assessed by pathological indicators such as CAG, IM, and Dys identified in stomach mucosa. **Table 3** enumerates various composite modeling methodologies and their corresponding model types. Combined modeling approaches enhance model stability and, to some extent, compensate for the limitations of single-factor models. They also take into account animal welfare concerns, making them better aligned with the 3R principles (Replacement, Reduction, and Refinement).Despite variations in modeling cycles and outcomes, potentially due to supplementary modeling techniques, medication compositions, and laboratory settings, the results remain significant references for researchers.

**Table 3:** Overview of Combination Modeling Protocols for Gastric Precancerous Lesions and Cancer.

MNNG Dose & Schedule	Co-Factors	Duration (Wks)	Model Outcome	Repeated verification	Animal strains
Free drinking water(200 μg/mL)	Irregular feeding	15 (88),16 (89) 24 (90)	IM + Dys	Have	Sprague-Dawley male rats (88, 90);Sprague-Dawley rats (89);
Free drinking water(150 μg/mL)	Weekly 0.1 mL 10% NaCl x 20w	28 (91)	Dys	Have	Sprague-Dawley male rats
Free drinking water(180 μg/mL)	Intragastric 2% Sodium Salicylate (1mL/100g)	24 (92)	CAG	Not	Wistar male rats
Free drinking water(200 μg/mL)	0.03% Ranitidine feed; Fasting (q/2d); Gavage 40% Ethanol (10 ml/kg/d)	26 (93)	CAG + IM + Dys	Not	Sprague-Dawley male rats
Intragastric Gavage (200 mg/kg/15d)	Gavage 40% Ethanol (1 mL/q3d) + Fasting (q/3d)	16 (94) 20 (95)	IM	Have	Wistar male rats
Free drinking water(170 μg/mL)	0.03% Ranitidine feed; Fasting (q/3d); Gavage 2% Sodium Salicylate	12 (96)	IM	Not	Sprague-Dawley male rats
Free drinking water(100 μg/mL)	Alternating gavage: 20% Ethanol & 50°C Hot Saline + Ranitidine (2.25g/L, 2mL/d); Irregular feeding	28 (97)	IM	Not	Sprague-Dawley male rats
Free drinking water(100 μg/mL)	0.3% Ranitidine feed; Fasting; Gavage 150g/L 56°C NaCl + 30% Ethanol	16-24 (98)	IM + Dys	Not	Wistar male rats

Although numerous studies utilize MNNG-induced precancerous lesions as their endpoint, the specific modeling periods vary significantly—ranging from 12 to 28 weeks. This variation is not merely random inconsistency, but rather a reflection of differences in model design, the dynamic progression of precancerous lesions, variations in experimental protocols, and subtle discrepancies in evaluation criteria. The lack of a standardized approach has led to the use of varying combinations and concentrations of N-nitroso compounds, making it difficult to critically compare conclusions across studies.Furthermore, pathological changes themselves vary in severity. Time points such as 12–16 weeks may capture the initial stages of precancerous pathways, emphasizing “mild” or “early” lesions, while time points around 24–28 weeks may approach the critical transition between precancerous lesions and neoplastic states, highlighting “severe” or “high-grade” pathology. However, most studies only report changes in pathological status without detailing the extent of these alterations, leaving readers confused when confronted with inconsistent results. This underscores an urgent need for greater data standardization.

MNNG is frequently utilized in conjunction with NaCl, ethanol, bile acids, ammonia water, ranitidine, or dietary modifications to reduce modeling duration and enhance efficacy. Zhu Y et al. given MNNG at a dosage of 200 mg/kg, every 15 days, in conjunction with 40% ethanol (1 mL) every 3 days, with a 3-day fasting regimen, successfully created a model of precancerous gastric lesions in rats during a 20-week period (95). ChunYue Yu et al. (99) employed a methodology that included ad libitum consumption of MNNG solution (100 μg/mL) and a diet supplemented with 0.05% ranitidine, alongside irregular feeding and administration of a 2% sodium salicylate solution (0.5 mL per 100 g body weight), to effectively create a gastric cancer model.

###### 4.1.1.2.1 NaCl

While it can not induce cancer, it can amplify the carcinogenicity of MNNG, simulating high-salt diets. Rats provided with 10% sodium chloride in their drinking water consume 1.2 to 1.5 times more than those without sodium chloride (100), and this increase is dose-dependent (101). Alongside the application of MNNG for modeling, the weekly administration of 0.1 mL of 10% sodium chloride solution effectively established the Dys model after 28 weeks (91, 102). Moreover, NaCl consumption can significantly mitigate the decrease in water intake induced by high-concentration MNNG solutions in animals. The factors contributing to NaCl’s role in tumorigenesis can be delineated as follows: 1) Decreases gastric mucus viscosity, compromising the mucosal barrier (99, 103); 2) Elevates ornithine decarboxylase (ODC) activity and replicative DNA synthesis (RDS), indicators of tumor promotion (101); 3) Augments lipid peroxidation associated with gastric epithelial proliferation (103, 104).

###### 4.1.1.2.2 Bile acids

Simulate harm caused by bile reflux. The incorporation of 0.2% taurocholic acid into the diet elevated antral tumor formation from 25% (with MNNG alone) to 72% (105). Various bile salts exert a stimulating influence on the stomach mucosa (106). Bile salts influence the ion channels of gastric mucosal cells, allowing hydrogen ions from the lumen to penetrate the mucosa, so compromising the stomach epithelial barrier and facilitating the absorption of possible carcinogens (107). Sodium deoxycholic acid is currently employed to replicate the irritative effects of bile reflux on gastric mucosa, diminish its barrier function, induce inflammatory responses, and facilitate the development of gastric cancer models.

###### 4.1.1.2.3 Ammonia water

Helicobacter pylori is designated as a Group I carcinogen associated with the onset of stomach cancer. This bacteria exhibits robust urease activity, converting urea in the stomach into ammonia. Intragastric gavage of Hp bacterial water can improve the success rate of model development (108, 109). Nonetheless, as HP is a biological pathogen, it necessitates a high degree of technical proficiency from laboratory professionals and particular environmental conditions. The application of ammonia water in modeling can replicate the high-ammonia conditions and harmful effects associated with H. pylori (Hp) infection, resulting in chronic gastritis. The amalgamation of ammonia water with MNNG can markedly elevate the occurrence of the model (63, 110). In trials, ammonia water is often provided freely at concentrations of 0.05% to 0.1% on fasting days.

###### 4.1.1.2.4 Ranitidine

Moreover, a low-acidic environment can expedite the advancement of stomach cancer (111). An elevation in gastric pH can augment the methylation of MNNG (112). Consequently, it is recommended to utilize the acid-suppressing medication Ranitidine together with modeling. It is essential to recognize that, given the half-life of Ranitidine, to maintain its efficacy in suppressing stomach acid secretion, it is often incorporated at a concentration of 0.03% to 0.05% into rat meal for ad libitum consumption and must be maintained in a dry, cool environment.

###### 4.1.1.2.5 Alcohol

An ethanol solution replicates the actual danger associated with alcohol consumption, a significant risk factor for stomach cancer, and can facilitate the dissolution of MNNG solutions. It is frequently employed in combination to augment MNNG consumption, with 20% glycolic acid markedly elevating both the occurrence and quantity of MNNG (113). Research indicates that ethanol concentrations of 11% can inhibit tumor formation (114). Elevated quantities of 20%-40% ethanol solution are generally delivered via gavage, necessitating vigilant monitoring of the animal’s condition to avert asphyxiation resulting from intoxication.

###### 4.1.1.2.6 Sodium salicylate

Prolonged use of non-steroidal anti-inflammatory medicines (NSAIDs) has been extensively researched for its enduring detrimental effects on the gastric mucosa. Sodium salicylate, a frequently utilized pharmaceutical in modeling protocols, can induce harm to vascular endothelial cells, resulting in an inflammatory milieu (115). It also impedes prostaglandin synthesis, compromising the protective barrier of the stomach mucosa. The standard dosage in modeling methods is 2% sodium salicylate. To augment its efficacy, it is frequently paired with an erratic diet. Following a day of fasting, the medication is delivered via gavage to enhance contact duration with the gastrointestinal mucosa.

###### 4.1.1.2.7 Others

Furthermore, the amalgamation of heated saline and intragastric gavage can replicate the irritation induced by high-temperature food on the gastric mucosa and is frequently employed in modeling procedures. An irregular diet, as a primary approach of dietary intervention, can elevate the likelihood of irregular eating patterns. The aforementioned conditions, in conjunction with substances like sodium salicylate and ethanol, can be amplified by doing stomach lavage post-fasting to augment their stimulating effects.

MIWA H et al. (116) documented the dynamic pathological alterations in the gastric mucosa of rats following unrestricted consumption of a 100μg/mL MNNG aqueous solution, which corresponds with the pathological course of gastric cancer as delineated by the Correa model. Kogure K et al. (64) discovered that MNNG delivery resulted in three phases of gastric mucosal alterations. The first stage is predominantly manifested as gastric mucosal injury and atrophy. By the eighth week, superficial erosion and significant atrophy were evident in the antral mucosa. By week 16, significant surface erosion and minor dysplastic glands were noted in the antrum. The second phase is characterized by intestinal metaplasia and dysplasia. Beginning in week 18, irregular atypical glands commenced development near the erosion edges and the pylorus. At week 24, the antral surface epithelium exhibited papillary hyperplasia, characterized by the presence of irregular and atypical glands. The final stage is gastric carcinoma. By week 26, hyperplasia of the surface epithelium and pyloric glands was noted in both the minor and larger curvatures of the antrum, with or without atypia. Between weeks 25 and 32, adenocarcinoma and spindle cell sarcoma were identified, alongside submucosal sarcomas exhibiting atypical gland invasion in the lesser curvature of the antrum. This aligns with prior research (117), which indicated that 12 weeks post-MNNG exposure, the mucosal layer of rat gastric tissue thinned, signifying superficial gastritis; after 24 weeks of MNNG treatment, the gastric mucosa displayed considerable inflammatory cell infiltration, diminished gland size, and reduced gland quantity, leading to a diagnosis of chronic atrophic gastritis (CAG); after 36 weeks of MNNG treatment, the gastric mucosa in the rats revealed inflammatory granulomas or ulcer-like lesions, with glands contracting due to vacuolar goblet cells, and the pathological alteration was atrophic gastritis with intestinal metaplasia (IM). Following 48 weeks of MNNG exposure, the glandular architecture was obliterated, and the pathology exhibited irregular morphologies. The peribasilar membrane is encircled by numerous inflammatory cells of diverse sizes, categorized as dysplasia (Dys). Qiu-yue Li and colleagues (118) discovered that following 12 weeks of MNNG exposure in rats, the stomach mucosal epithelial cells were partially substituted by intestinal-type epithelial cells. After 20 weeks, the stomach mucosa commenced atrophy, progressively deteriorating to moderate or severe stages. The detailed characteristics of the three stages of gastric mucosal alterations induced by MNNG administration are summarized in **Table 4**.

**Table 4:** The stages of gastric mucosal lesion development induced by MNNG.

Dosage	Route of Administration	Time	Pathological alterations	Time	Pathological alterations	Time	Pathological alterations	Quote
100 mg/l	via Drinking Water	2M	Atrophy and erosion of gastric mucosal tissue	4M	Mild intestinal metaplasia, focal degenerative changes in mucosal glands, marked lymphoid infiltration, and granuloma formation	6-8M	Adenomatoid proliferation, poorly differentiated adenocarcinoma, mucosal atrophy with stromal fibrosis and hyalinization	(119)
50-83 μg/ml	via Drinking Water	8W	Erosion and atrophy of the mucosal surface in the antral cavity	16-24W	Glandular dysplasia, papillary hyperplasia of the surface epithelium, and irregular atypical glandular hyperplasia	25-31W	Atypical hyperplasia of surface epithelium and pyloric glands, adenocarcinoma, spindle cell sarcoma, signet-ring cell carcinoma (SRCC), and submucosal sarcoma with atypical glandular infiltration	(64)
100 mg/l	via Drinking Water	12-24W	Thinning and atrophy of the gastric mucosal layer, with reduced number and size of glands	36W	Inflammatory granulomas or ulcerative lesions with intestinal metaplasia in the gastric mucosa	48W	Irregular glandular architecture with cellular dysplasia	(117)
100-200μg/mL	via Drinking Water	1M	Inflammatory changes in the gastric mucosa	3M	Gastric mucosal epithelial cells were partially replaced by intestinal-type epithelial cells in rats	5M-8M	Rod-shaped atypical proliferating cells were observed in the gastric mucosa, exhibiting hyperchromatic and enlarged nuclei, and an increased nuclear-to-cytoplasmic ratio	(118)

A comparison of the characteristics of different drug administration methods is presented in **Table 5**. The selection of a modeling approach involves multi-dimensional trade-offs. In terms of organ specificity, there is currently no ideal tumor model that fully meets all criteria. Compared to other methods, the gavage + combined modeling approach may represent a preferable option. However, from the perspective of animal welfare and simulating the natural progression of disease in humans, free drinking + combined modeling might be a more desirable alternative. The choice of an appropriate modeling strategy should be comprehensively weighed according to research objectives. Given the substantial burden of cancer worldwide, investigating the mechanisms and therapeutic targets of precancerous lesions and states has become a research focus,areas where combined modeling approaches are widely applied.

**Table 5:** Comparison of characteristics of different administration methods in MNNG gastric cancer related models.

Characteristic	Free Drinking	Intragastric gavage	Intragastric gavage+Composite modeling	Free Drinking+ Composite modeling
Core Features	Chronic, continuous, low-dose exposure; mimics natural process	High-dose concentrated administration; focuses on gastric exposure	Rapid initiation + multiple promoting factors;Efficient model establishment	Chronic initiation + multiple promoting factors;Balances efficiency and natural exposure
Operational Difficulty	Low	High	Higher	Moderate
Organ Sensitivity	Poor; broad exposure, may induce esophageal and small intestinal tumors	Moderate; may induce forestomach tumors	Probably High; combined factors increase incidence of glandular gastric tumors	Probably High; combined factors enhance glandular gastric specificity
Common Target Models	Tumor and precancerous models	Tumor and precancerous models	Relatively short Precancerous models	Relatively short Precancerous models
Model Stability/Success Rate	Unstable	Relatively stable	Stable	Possibly better than single method, but slightly inferior to gavage + combined method
Modeling Cycle	Relatively Long	Relatively Long	Relatively short	Relatively short
Animal Welfare	Moderate;No procedural stress but long duration and high multi-tumor burden	Poor; high procedural stress, acute injury risk, and high suffering	Moderate (optimizable); short cycle, good uniformity, reduces animal use	Better;Avoids gavage stress, shorter cycle than single-factor administration
MNNG Intake Control	Poor	Good	Good	Moderate; multiple factors can synergistically increase MNNG intake
MNNG Environmental Pollution	High; long-term contamination via water bottles and bedding, high risk to staff	Low;MNNG prepared freshly, minimal contamination, easy to disinfect	Low;MNNG prepared fresh, minimal contamination, easy to disinfect	High; long-term contamination via water bottles and bedding, high risk to staff
Others	Requires high storage conditions for MNNG	–	–	Requires high storage conditions for MNNG
Main Advantages	Closest to natural process; no procedural stress	Accurate dosing; low contamination	Good lesion uniformity; high organ specificity; high research efficiency	Balances efficiency and animal welfare; avoids procedural stress; good lesion uniformity
Main Disadvantages	Unstable model formation; low efficiency; multi-organ damage; environmental pollution	High procedural stress; may induce forestomach tumors; poor animal welfare	Complex operation; high requirements for experimental design and management	Still poses environmental risk; more complex than single-factor methods
Suitable Applications	Chronic toxicity; natural progression of gastric cancer	Inducing stable measurable solid tumors; suitable for anti-cancer drug efficacy studies	Mechanisms of precancerous lesions; screening of chemopreventive drugs; anti-cancer target research	Multi-factor synergistic carcinogenesis mechanisms; mimics natural human environmental lesions with emphasis on animal welfare

##### 4.1.1.3 Similarities and differences between human andMNNG-induced gastric cancer

From an etiological perspective, MNNG can mimic the damage caused by nitrites to the gastric mucosa, which is similar to nitrite-induced human gastric cancer (120). Moreover, when combined with factors such as ethanol, high salt intake, dietary habit changes, nonsteroidal anti-inflammatory drugs (NSAIDs), and Helicobacter pylori infection, MNNG can simulate a more complex environment resembling human tumor development. After MNNG intervention, rats develop inflammatory responses in the gastric mucosa, with pathological manifestations similar to human intestinal-type gastric cancer (116, 117). The histological progression follows the pattern of “chronic superficial gastritis -chronic atrophic gastritis - intestinal metaplasia - dysplasia - tumor,” making it a commonly used model for studying precancerous lesions.

Mutations in genes such as Bcl-2, COX-2, H-ras, and p53 have been documented in both human and MNNG-induced gastric tumors (121). However, the expression of oncogenes such as Ki-ras and β-catenin does not increase in MNNG-induced rats (122), suggesting that these genes are not major drivers in MNNG-induced rat gastric cancer or in human gastric cancer. Additionally, no microsatellite instability (MSI) is observed. Many genes involved in immune responses are upregulated, along with genes related to extracellular matrix (ECM) remodeling, while genes associated with gastric differentiation are downregulated (123). The upregulation of immune/inflammatory response genes is consistent with findings in human gastric cancer. Thus, based on molecular expression profiles, MNNG may serve as a suitable model for differentiated gastric cancer (123).

However, the molecular mechanisms by which MNNG mimics human gastric cancer have certain limitations. While p53 mutations occur in 36–40% of differentiated human gastric cancers, their incidence in rats is very low (122, 124). Moreover, no common human gastric cancer mutations, such as Ki-ras mutations, K-sam amplification, or c-erbB-2 gene amplification, have been detected in MNNG-induced rats (123). The cell cycle regulatory gene Cyclin D1, which is typically upregulated in human gastric cancer, is downregulated in rat models, showing an opposite trend. Genes associated with lymph node metastasis in human gastric cancer (e.g., Rbp4, Igf2, Fn1) are not significantly upregulated in rats, indicating a lower potential for lymph node metastasis in MNNG-induced rat gastric cancer. Additionally, promoter CpG island (CGI) methylation of tumor suppressor genes such as CDH1 (E-cadherin), CDKN2A (p16), MLH1, and RASSF1A (19), which is commonly reported in human gastric cancer, has not been observed in MNNG-induced rats.

Overall, while the disease background of MNNG-induced tumors is relatively simple and may not fully replicate the complex microenvironment of tumor development, MNNG remains a valuable chemically-induced model for studying human differentiated gastric cancer in terms of epigenetics, histopathological features, and molecular immune responses.

#### 4.1.2 Animal models associated with colon cancer

MNNG is a powerful topical carcinogen commonly employed to cause colon cancer, namely well-differentiated adenocarcinomas, situated in the distal colon (125, 126). Intrarectal Instillation: This is the conventional technique, facilitating targeted induction in the distal colon and rectum.Sterile circumstances expedite tumor development and elevate incidence relative to typical surroundings (127, 128). So BT et al. (129) developed colon cancer in all 30 rats with intrarectal administration of MNNG (2 mg/kg) biweekly for 250 days. Kannen V and Frajacomo FT (130, 131) administered four doses of MNNG (5 mg/mL, 0.5 mL/dose) biweekly for two weeks, resulting in the induction of colon cancer within 8-10 weeks. Weekly intrarectal administration of MNNG (1-3 mg/rat) for 20 weeks in male F344 rats resulted in colorectal cancer, comprising 57% adenomas and 43% adenocarcinomas (132, 133). MNNG induces pre-neoplastic lesions such as aberrant crypt foci (ACF) (134). Histologically, it induces goblet cell depletion and lymphocytic infiltration (135). We observed that F433 mice (98) exhibited a significantly higher mutagenesis rate of 89% compared to other models using the same modeling method, which may be directly attributed to differences in animal strains.

MNNG, a direct carcinogen that does not necessitate metabolic activity, elevates p53 protein levels with sustained exposure, signifying active epithelial proliferation (98). Intrarectal injection preferentially promotes cancer at the site of exposure, closely resembling natural development. Intrarectal administration of MNNG represents a stable method for inducing colorectal cancer, with higher total dosage correlating to increased tumor induction rates and correspondingly elevated mutagenesis, as detailed in **Table 6**. Nonetheless, the modeling cycle is protracted, and precisely determining the dosage for rectal delivery poses difficulties. It is essential to maintain the animals in an inverted position for one minute post-administration to avert the reagent from reverting to the anus and compromising the modeling effect (145, 146).

**Table 6:** Overview of Intrarectal MNNG Protocols for Colon Cancer Models.

MNNG dosing groups	MNNG Dose & Schedule	Co-Factors	Outcome/Success Rate	Repeated verification	Animal strains
Low- dosage	Single dose: 8 mg/mL,0.5 mL/dose (Total 4 mg)	0.9%Saline	25% (136, 137)	Have	Donryu female rats (136);Charles River CD-Fischer rats (137)
	4 doses: 5 mg/mL, 0.5 mL/dose, twice weekly x 2w (Total 10 mg)	None	ACF by 10w (138); Tumors in 80% by 30w (134)	Not	Sprague–Dawley male Rats (138);Wistar male rats (134)
	4 doses: 4 mg/mL, 0.5 mL/dose, twice weekly x 2w(Total 8 mg)	0.9%Saline (135, 139);None (126, 140);	67% (139); 89% (126); 60% by 20w (140); 60% by 24w, 67% by 48w (135)	Partial verification	Sprague–Dawley male Rats (135, 139, 140);F344 female rats (126);
Middle-dosage	Dose escalation: 1 mg/wk (3w), 2 mg/wk (6w), 3 mg/wk (11w), (Total 48mg)	0.9%Saline	100% tumor incidence (Both in sterile & conventional) (141)	Not	CD Fischer female rats
	Daily: 1 mL of 2.5 mg/mL x 14d (Total 35 mg)	None	40% by 20w, 80% by 40w (142)	Not	Wistar male rats
	Daily:0.5ml of 2.5mg/ml x 32d (Total 40 mg)	None	76% (143)	Not	Donryu female rats
	Twice weekly x 53w: 0.5 mL of 1.25 mg/mL (Total 66.25 mg)	None	87% (144)	Not	female inbred strain-2 guinea pigs
High- dosage	Three times weekly x 25w: 2 mg/kg (Total 150 mg/kg)	Not mentioned	97% tumors observed between days 250-356 (127)	Not	Not mentioned

#### 4.1.3 Animal models associated with esophageal cancer

Esophageal cancer (EC) ranks as the eighth most prevalent cancer worldwide and the sixth in terms of death, with esophageal squamous cell carcinoma (ESCC) being the predominant variant (147). Exposure to nitrosamines constitutes a substantial risk factor for the development of esophageal squamous cell carcinoma (ESCC) (148), and nitrosamine chemicals behave as principal chemical carcinogens in esophageal cancer models. Suizhi Cheng’s team (11) discovered that MNNG can activate NF-κB, leading to the upregulation of inflammatory markers IL-6, IL-8, and TNF-α, which induces esophageal inflammation in SD mice. Moreover, MNNG can induce the proliferation of squamous epithelial cells in the esophageal mucosa of rats and promote the malignant transformation of human esophageal epithelial Het-1A cells (149). MNNG demonstrates heightened sensitivity to adenocarcinoma. N. Yioris et al. (150) injected 5.0 mg/kg of MNNG to the esophagus of 30 mice during a duration of 37 weeks. The research revealed that 11 animals in the cohort acquired stomach adenomas, 2 animals got esophageal squamous cell carcinoma, and 1 animal developed colon adenocarcinoma. Furthermore, five instances of hepatic cystadenoma and one instance of esophageal keratinizing papilloma were noted, indicating that MNNG carcinogenesis may exhibit non-specific traits for target organs.

The manifestation of EC is a complex process that encompasses both environmental and genetic influences. MNNG can replicate the external environmental elements associated with the progression of esophageal cancer. Moreover, while oral administration is the predominant technique for inducing esophageal cancer, MNNG may interact with neighboring organs, including the stomach and small intestine, resulting in carcinogenic effects. Consequently, it may serve as a co-inducer of esophageal and gastric cancer. Research conducted by Mamdooh H Ghoneum et al. demonstrates that MNNG serves as a model for esophageal and gastric adenocarcinoma, revealing that esophageal tissue pathology predominantly presents as squamous cell carcinoma, whereas gastric tissue pathology following carcinogen exposure displays glandular dysplasia and adenocarcinoma (151). Nonetheless, the incidence of esophageal cancer produced by Methyl benzylnitrosamine (NMBA) can attain 100% (152). MNNG exhibits inferior organ specificity compared to the widely utilized chemical inducer NMBA in the EC model. In practical application, it does not precisely trigger particular disease types or target organs, and is primarily utilized to provoke damage and malignant transformation of esophageal epithelial cells.

### 4.2 MNNG in other system models

#### 4.2.1 Animal models associated with lung cancer

Lung cancer is the malignancy with the greatest global mortality rate, and adenocarcinoma is one of its most common histological subtypes (1). MNNG can be employed to create lung cancer models. Research demonstrates that intravenous administration of MNNG can provoke the onset of lung cancer in animals (153). Lin Deng et al. (154) developed an early-stage lung adenocarcinoma (LAC) model by subcutaneously delivering a 0.4 mg MNNG solution weekly for four weeks. Tumor development was observed in all 10 animals, with micro-CT identifying a total of 231 tumors, all histologically verified as LAC. Additionally, Yan Ping Xie et al. (155) administered 0.4 mg of MNNG subcutaneously into the dorsal area of forty KM mice, once weekly for four successive weeks. The mice were categorized into four groups (n=10 per group), and tissue samples were obtained at 14, 18, 24, and 28 weeks, respectively. The research indicated that the adenomatous proliferation of cancer cells intensified over time, and the overall tumor count had a positive association with time. At week 28, the mean tumor count per mouse was 10.00 ± 5.64. MNNG exhibits significant carcinogenic properties and is frequently employed to produce early forms of lung cancer. Intravenous injection can effectively produce lung cancer in rats, however the mortality rate is elevated. Conversely, subcutaneous injection is more secure. MNNG influences lung tissue by absorption into the bloodstream and systemic circulation. This technique is straightforward to execute, maintains the integrity of lung tissue without harm, and prevents the introduction of confounding variables such as infection. In addition to localized tumors, low-dose repeated administrations can selectively generate lung tumors while sparing other organs from tumor development. Consequently, MNNG functions as a comparatively optimal drug for the induction of lung cancer models.

#### 4.2.2 Animal models associated with endometrial cancer

Endometrial cancer is the predominant malignancy of the female reproductive system, with a persistent upward trend in incidence and a rising prevalence among younger women (156). MNNG, a powerful carcinogenic mutagen, is utilized to create endometrial cancer models (157, 158). MNNG enhances AKT phosphorylation and PI3K activation through TGF-β activation, hence augmenting endothelial cell invasiveness (159). T. Tanaka et al. (158) showed through studies the impact of varying dosages, administration routes, and cycles on the experimental outcomes. It was found that intrauterine injection can induce endometrial adenocarcinoma and other uterine neoplasms. Elevated dosages may induce pronounced local effects leading to tissue damage and hindered tumor development, albeit potentially raising tumor incidence. Cervical and vaginal tumors are the primary focus of vaginal administration, providing a valuable experimental model for investigating the prevalence of uterine cancer. Pakkiri Bhavani et al. (157) employed cotton balls saturated with MNNG (150 mg diluted in 0.2 ml of olive oil) for vaginal retention biweekly in albino female Wistar rats. Currently, intracavitary injection and the placement of absorbent cotton balls into the vagina are the predominant techniques for delivering MNNG. Vaginal delivery simulates a natural infection or local exposure, whereas intrauterine injection may exert a more immediate impact on uterine tissue. Among these methods, the intravaginal approach with saturated pellets is most commonly employed in model establishment.

#### 4.2.3 Animal models associated with liver cancer

MNNG is categorized as a non-hepatocarcinogen and is rarely utilized in the development of liver cancer models. Chemical inducers including aflatoxin B1 (AFB1), carbon tetrachloride (CCl_4_), and diethylnitrosamine (DEN) are frequently employed in current research. MNNG is sometimes co-administered with several recognized carcinogens to improve the effectiveness of hepatocellular carcinoma induction or to expedite modeling deadlines. Research indicates that MNNG can activate oxidative stress responses, accelerate aberrant cell proliferation, and serve as an inducer of the initial phase of hepatocellular carcinoma (160). Prior studies have shown that administering 80 mg/kg MNNG for seven weeks can precipitate liver cancer when combined with CCL4 or partial hepatectomy (PH), significantly augmenting the quantity of glutathione S-transferase placental (GST-P) positive hepatocyte foci (161). Research by S.L. Herren et al. indicates that prolonged exposure to 0.005% MNNG in drinking water does not exhibit carcinogenic activity specifically affecting the liver (162). T. Ogiso et al. assert that non-hepatocarcinogens can solely generate tumors in certain target organs, shown by EHBN-induced bladder cancer and MNNG-induced gastric adenocarcinoma, without exerting a major promotional influence on the development of hepatocellular carcinoma (163). The origin of this paradoxical behavior may be attributed to variations in dosage and cycles, potentially elucidating why the carcinogenic effects have yet to materialize.

#### 4.2.4 Animal models associated with skin

Skin tumors represent one of the most prevalent malignant neoplasms in humans, encompassing a developmental process characterized by initiation, promotion, and advancement (138, 139). MNNG is frequently employed as an initiator in skin tumor models and can induce irreversible genetic alterations.

Investigation conducted by I. Rehman et al. proposes that MNNG facilitates the development of skin tumors by generating mutations in codon 12 of the Ha-ras and Ki-ras oncogenes (140). J F O’Connell et al. assert that MNNG functions as both a tumor initiator and a tumor promoter (141). The tumorigenicity of MNNG significantly escalates during the dosage range of 0.5 - 5.0 μmol when employed as a full carcinogen. A dose range of 0.1 to 2.0 μmol can induce papilloma formation as an initiator, however elevated doses of the promoter diminish papilloma development. As a promotional agent, MNNG dosage is positively correlated with the incidence of cancers.

G J Patskan et al. (142) administered 2 μmol of MNNG topically to the skin of mice. With the prolongation of the treatment time, the incidence of skin malignancies in the mice progressively escalated, and in the advanced stages, 20% of the mice had lung metastases. The carcinogenic potential of MNNG in eliciting skin cancer seems plausible, and the method of topical delivery is straightforward and expedient. The dose-effect relationship curve of MNNG is intricate, with excessive doses potentially elevating mortality. Additionally, in contrast to the commonly employed two-stage carcinogenesis protocol utilizing DMBA (7,12-dimethylbenz[a]anthracene) as the initiator and TPA (12-O-tetradecanoylphorbol-13-acetate) as the promoter, MNNG exhibits reduced specificity.

### 4.3 Multi-organ characteristics and optimization strategies of MNNG in different models

#### 4.3.1 The relative specificity of MNNG in gastric cancer-related models

Previous studies have indicated that MNNG exhibits relative specificity in inducing gastric cancer models (164). Following oral administration, the stomach demonstrates the highest level of DNA alkylation. This may be attributed to MNNG’s stability in the acidic gastric environment and the fact that the stomach is exposed to the highest concentration of the compound (165). Studies have shown that the activity of DNA repair enzymes such as MGMT is relatively low in the gastrointestinal tract, leading to the accumulation of alkylation damage (166, 167).

#### 4.3.2 MNNG’s multi-organ properties

As discussed previously, we have examined the application of MNNG in other organs, which sufficiently demonstrates its carcinogenic potential across multiple organs.As a direct alkylating agent, MNNG induces tissue DNA damage without requiring bioactivation (16). It exhibits first-pass effects and localized exposure characteristics, with its organ specificity strongly dependent on the route of administration (168). Currently, MNNG is most commonly used in gastric cancer and pre-gastric cancer models, administered by drinking water and gavage.As a hollow organ connected to multiple parts of the digestive tract, the stomach allows MNNG solution to come into contact with various organs when administered in drinking water. After absorption through the stomach and small intestine, MNNG can be transported to other tissues and organs via systemic circulation and enterohepatic recirculation (169). Studies have reported the development of esophageal sarcomas and tumors in the stomach, liver, and jejunum following MNNG exposure in modeling experiments (150). Research by Mamdooh H. Ghoneum et al. (151) established a comorbid model of esophageal and gastric cancer using MNNG solution, further confirming its ability to induce multi-organ injury.

#### 4.3.3 Organ-specific optimization of MNNG in model applications

Nonetheless, MNNG is not always the optimal choice for various cancer models. Prioritization of organ-specific carcinogens: When conditions permit, agents with high organotypic specificity should be prioritized. For instance, 4-nitroquinoline 1-oxide (4NQO) and N-nitrosomethylbenzylamine (NMBA) show stronger specificity for esophageal cancer induction (170, 171); diethylnitrosamine (DEN) is more suitable for liver cancer models (172, 173); and 1,2-dimethylhydrazine (DMH) and its metabolite azoxymethane (AOM) are preferred for modeling colon cancer (133, 174). In specific organ contexts, these agents may outperform MNNG in terms of modeling efficiency, duration, and specificity. A comparative summary is provided in **Table 7**.

**Table 7:** Organ-specific evaluation of different modeling drugs.

Specific organs	Reagent	Organ Specificity Compared to MNNG	Mutational Signatures	Translational Relevance	Quote
Gastric Cancer	MNNG	high	The formation of DNA adducts O^6^-meG and N^7^-meG induces G:C to A:T transition mutations,; mutations in key genes such as p53	The tumors induced by mimicking dietary nitrosamine intake closely recapitulate the pathological morphology and molecular alterations of human gastric cancer.	(175, 176)
	Mnu	intermediate	The O^6^-meG and N^7^-meG lesions mediate and predispose to G:C to A:T transition mutations; Induction of DNA hypomethylation in the MGMT promoter region	Mimicry of dietary nitrosamine exposure	(18, 177, 178)
Colon Cancer	AOM	high	The reactive intermediates (such as diazomethane) produced during AOM metabolism can directly alkylate DNA, resulting in O^6^-meG-mediated G:C > A:T transition mutations; Mutations in key genes such as K-ras and p53.	Simulating the multistage evolution process of human sporadic CRC and its key molecular features	(179–181)
Esophageal Cancer	NMBA	high	Point mutation at codon 12 of Ha-ras (GGA→GAA, Gly→Glu); Mutations in exons 5 and 7 of p53	The lesion sequence closely resembles that of human ESCC	(182–184)
Lung Cancer	NNK	high	Activation of the KRAS proto-oncogene, with G12D (GGA→GAC, Gly→Asp) and G12C (GGA→TGC, Gly→Cys) mutations; Formation of O^6^-meG, leading to G:C→A:T transition mutations	Simulating tobacco smoke-induced tissue lesions in humans	(185–187)
Liver Cancer	DEN	high	Mutations include C→A transversions; C→T and T→C transitions.; Mutations in the CTNNB1 (β-catenin) gene and TP53 gene	Simulating the multistage pathological evolution process of human Hepatocellular Carcinoma	(177, 188, 189)
Skin Cancer	DMBA	high	A highly specific A→T transversion at codon 61 of the HRAS gene (Q61L, Gln→Leu). Mutations in the Hras, Kras, Rras2, and Trp53 genes	It is widely used to screen and validate a large number of natural or synthetic chemopreventive agents, evaluating their inhibitory effects at different stages of carcinogenesis (initiation, promotion, progression)	(190, 191)

In models of tumors outside the digestive tract, although MNNG possesses carcinogenic potential, it is not the most commonly used or specific mutagen. In contrast, MNNG demonstrates a relatively high application rate and specificity in inducing gastric cancer. Nevertheless, off-target tumor development remains a concern (6, 86, 192, 193).Consequently, a significant scientific challenge exists in improving the efficacy of these medicines to selectively produce malignancies in the designated organ.While ensuring modeling efficiency, there is an urgent need to explore how to optimize the modeling approach to achieve higher specificity, better model stability, and more comprehensive animal welfare protection.

When experimental funding allows, the use of gene knockout models in combination with MNNG can be employed to enhance organ sensitivity (194). Optimizing the modeling timeline by selecting MNNG as the initiating factor and other modeling methods as promoting factors can not only more classically simulate different stages of carcinogenesis but also avoid the drawback of long-term MNNG application inducing multi-organ tumors (195). If a tendency for multi-organ tumor development is observed during the process, the promoting factors can be withdrawn to create a window period for further investigation. Furthermore, monitoring during the process should be optimized.Incorporating contemporary technologies is essential. Employing periodic blood tests to monitor early tumor biomarkers and evaluate potential liver damage induced by MNNG (152, 153), facilitating prompt modifications to dosage and scheduling. The endoscopic examination facilitated rapid evaluation, enabling direct visualization to detect model advancement and mitigate excessive exposure (154, 155). Multi-Parametric MRI (MP-MRI) serves as an effective instrument for the detection, localization, and characterization of primary tumors in experimental animals (192).CT and PET are also considered to be effective monitoring methods (196).

Employ more precise drug administration methods to avoid off-target effects: Utilize localized administration techniques such as intragastric infusion or other targeted delivery approaches to enhance specificity.Utilizing these strategies can capitalizing on specific agents where feasible, precisely regulating dosage and administration for multi-organ carcinogens, and employing advanced monitoring facilitates the early identification of carcinogenesis and metastasis. This proactive strategy enables researchers to intervene and ultimately reduce the incidence of off-target cancers in other organs.Promote the establishment of standardized model protocols.Animal models exhibit intrinsic limits. Literature reviews indicate considerable variability in model development, with the absence of essential experimental data significantly compromising the integrity of model assessments and study findings. A prior study of more than 250 publications linked to animal models revealed that less than 60% of the articles specifically documented at least three animal characteristics (gender, strain, weight, age) and the number of animals utilized (197). Significantly, blinded experiments were exceedingly uncommon. Document and publicly disclose detailed experimental parameters, including animal age (in weeks), body weight, housing conditions, MNNG concentration, administration method, MNNG supplier and storage conditions, as well as diet and water specifications. Define clear criteria for successful model establishment, using pathological findings as the gold standard and categorizing lesions based on severity (198). Transparently report negative outcomes: Accurately document and publish animal mortality rates, incidents of multi-organ tumors, and cases of acute intoxication during experiments. Avoid redundant experiments by maximizing the utility of existing data, ensuring its value is fully leveraged.

## 5 MNNG defects and optimization

MNNG is classified as a Group I carcinogen, exhibits both cytotoxic and genotoxic effects (199, 200). Its cytotoxicity may arise through non-mutagenic mechanisms, and the dominant outcome—cell death or mutagenic carcinogenesis—largely depends on exposure concentration and duration (201). Therefore, balancing MNNG-induced mutagenesis and cell death remains a critical challenge. Additionally,MNNG demonstrates dose- and time-dependent hepatotoxicity (202). The severe cytotoxicity, genotoxicity, and hepatotoxicity of MNNG can lead to premature death in experimental animals,for instance, due to acute toxicity rather than cancer development,which complicates the assessment of its carcinogenicity. Additionally, direct exposure to MNNG may pose risks to laboratory personnel and the environment. Optimizing dose-dependent threshold effects is essential to maximizing tumor induction efficiency while minimizing animal mortality.The scientific value of MNNG must be carefully weighed against its potential hazards to ensure it is utilized in a safe and controlled manner, maximizing its research benefits while minimizing harm. This balance remains an important topic for further discussion.

Another limitation of MNNG lies in the difficulty of fully replicating the natural human carcinogenic process through its administration route and dosage. Except for occupational exposures, human contact with carcinogens is typically localized, long-term, and low-dose. In addition to nitrosamines, factors such as Hp infection also contribute to carcinogenesis. Therefore, mechanistic insights or preventive strategies derived solely from MNNG-based models may have limited extrapolation to human contexts.

The localized effects of MNNG contribute to its limited organ specificity, making it challenging to establish pure and organ-specific tumor models. While tumors form in target organs such as the stomach (e.g., gastric adenocarcinoma), concurrent neoplasms or damage often occur in other sites, including forestomach, esophagus, lung adenocarcinoma, liver injury, or colonic lesions. The coexistence of multiple tumor types alters the host’s immune status and metabolic environment, complicating the tumor microenvironment of interest. This complexity compromises the precision, reproducibility, and reliability of intervention studies. Moreover, multi-tumor incidence increases suffering in experimental animals. Nevertheless, optimizing time–dose thresholds and employing combination modeling approaches may enhance its relative specificity and reduce off-target organ damage. Additionally, organoid models—which mimic the complex crosstalk among diverse cell types within tissues—may help overcome the nonspecificity associated with MNNG exposure (5, 199).

Secondly, the environmental and ecotoxicological impact of MNNG represents a critical concern. Improper storage, handling, or disposal during experimental procedures may lead to MNNG leakage into the environment. Highly water-soluble, MNNG can enter water cycles and soil systems through sewage and waste disposal. As a genotoxic and cytotoxic agent, it induces DNA mutations and may bioaccumulate, thereby threatening ecological stability.

Due to the toxic nature of MNNG, its potential for environmental contamination, and the specific requirements of tumor research, it is necessary to optimize animal welfare protection policies during experiments. Rigorous ethical review is required. Prioritizing human safety involves strict toxic waste disposal procedures for MNNG to prevent environmental pollution. Researchers must be fully informed of MNNG’s carcinogenic risks and implement rigorous protective measures to minimize exposure. When handling the agent, personnel must wear masks, gloves, head coverings, and goggles if necessary. They should also be trained in the safe preparation, administration, and disposal of MNNG-contaminated items such as water bottles and cages. Laboratories must classify and label MNNG with the correct hazard identifiers, and experimental waste containing MNNG must be treated as high-risk toxic waste.Personnel must receive advance training to master key techniques including oral gavage, rectal administration, and vaginal inoculation to avoid causing unnecessary suffering to animals due to operational errors (203). Administration strategies should be optimized based on target organs, and the maximum tolerable dose of the carcinogen should be determined. Continuous monitoring and timely adjustments to the experimental protocol are essential. Analgesics must be administered when necessary to alleviate pain, and all observations should be accurately recorded.Humane endpoints must be established: for animals bearing a single tumor, the average diameter should generally not exceed 1.2 cm in mice or 2.5 cm in rats—or 1.5 cm and 2.8 cm, respectively, under certain conditions. If multiple tumors are present (e.g., on contralateral flanks), the size of each should be proportionally smaller and must not exceed the maximum burden of a single tumor (196). Animals showing signs of cachexia or a weight loss exceeding 20% of normal adult body weight require immediate intervention. No animal should be allowed to die naturally from suffering.

## 6 Discussion

Spontaneous animal tumor models provide a notable advantage: their genesis and course closely resemble human tumors, displaying similar histological complexity and heterogeneity to true human malignancies (192, 204). Despite certain limitations, MNNG remains widely used as a straightforward and effective chemical carcinogen in studies investigating spontaneous tumors and precancerous lesions.Future efforts should focus on enhancing the organ-specific sensitivity and stability of the MNNG-induced model, mitigating associated risks in its application, reducing the number of experimental animals used, and minimizing suffering throughout the experimental process.

Nevertheless, these enhancements are inadequate for progressing model development. Researchers must offer experimental results with thorough and comprehensive information to ensure reproducibility in future studies and optimize model utilization. Considering that laboratory animals bear considerable scientific obligations and utilize important research funding (205), the lack of high-fidelity, repeatable models will hinder contemporary scientific advancement. We can only honor the lifelong commitment of experimental animals to scientific progress by maximizing their value.
